# Framework for environment perception: Ensemble method for vision-based scene understanding algorithms in agriculture

**DOI:** 10.3389/frobt.2022.982581

**Published:** 2023-01-12

**Authors:** Esma Mujkic, Ole Ravn, Martin Peter Christiansen

**Affiliations:** ^1^ Automation and Control Group, Department of Electrical and Photonics Engineering, Technical University of Denmark, Kongens Lyngby, Denmark; ^2^ AGCO A/S, Randers, Denmark

**Keywords:** environment perception, ensemble models, object detection, anomaly detection, semantic segmentation

## Abstract

The safe and reliable operation of autonomous agricultural vehicles requires an advanced environment perception system. An important component of perception systems is vision-based algorithms for detecting objects and other structures in the fields. This paper presents an ensemble method for combining outputs of three scene understanding tasks: semantic segmentation, object detection and anomaly detection in the agricultural context. The proposed framework uses an object detector to detect seven agriculture-specific classes. The anomaly detector detects all other objects that do not belong to these classes. In addition, the segmentation map of the field is utilized to provide additional information if the objects are located inside or outside the field area. The detections of different algorithms are combined at inference time, and the proposed ensemble method is independent of underlying algorithms. The results show that combining object detection with anomaly detection can increase the number of detected objects in agricultural scene images.

## 1 Introduction

In the next few decades, agricultural production and the pressure to meet food demand are expected to rise due to global population growth. As a result, global food production needs to increase by 60 per cent by 2050 to feed the growing population (Alexandratos and Bruinsma, 2012). Technological development will play a vital role in the more efficient use of natural resources and sustainable agricultural practices (FAO, 2018). As the focus on farming productivity and efficiency has grown over the past few decades, more sophisticated and intelligent agricultural machinery has been developed. With the technological advancements, farming solutions will evolve from providing decision support to vehicle operators to in-field supervision of unmanned vehicles and eventually to fully autonomous vehicles.

The development of self-driving agricultural vehicles has attracted attention in the last few decades (Case, 2016; Kubota, 2017; New Holland Agriculture, 2017; YANMAR AGRIBUSINESS, 2019; AGROINTELLI, 2020; ASI, 2020). Nevertheless, the current self-driving agricultural vehicles have limited environment perception capabilities. Agricultural fields are dynamic and unstructured environments that change throughout different cycles. For autonomous agricultural vehicles to meet the safety requirements, they must be equipped with robust and real-time environment perception algorithms. Such an environment perception system needs to extract relevant knowledge from the environment and provide a contextual understanding of the vehicle’s surroundings.

In the last few decades, obstacle detection for agriculture attracted the attention of researchers. Several approaches to obstacle detection in agriculture leverage homogeneous characteristics of the agricultural field to detect obstacles in the foreground. Ross et al. (2014) proposed an anomaly detection system for obstacle detection in the agricultural field. The anomalies are identified in images, and stereo-matching is used to determine the obstacle’s location. An approach for detecting static and dynamic obstacles in the agricultural environment is proposed by Campos et al. (2016). In the approach, spatial-temporal analysis is applied to a video sequence. The obstacles are detected based on colour and texture features, while temporal information is used to capture the object’s movement. This method is able to extract obstacle areas from the image background and discriminate between static and non-static obstacles. The work presented in Christiansen et al. (2016) combines convolutional neural network (CNN) and background subtraction algorithms for anomaly detection in grass fields. This approach showed success in detecting heavily occluded, distant and unknown objects.

Another approach combines information obtained from image data with depth information to detect obstacles and produce obstacle maps in 3D. Korthals et al. (2018) proposes a multi-modal approach to detecting and mapping static and dynamic obstacles for grass-mowing operations. Four detection algorithms are applied to data from the stereo camera. Locally Decorrelated Channel Features (LDCF) (Xu et al., 2021) and You Only Look Once (YOLO) (Redmon et al., 2016) are applied for object detection, DeepAnomlay (Christiansen et al., 2016) is applied for anomaly detection, and fully convolutional network (FCN) (Long et al., 2015) is used for semantic segmentation. The algorithms are trained on publicly available datasets by remapping general classes of objects to classes relevant to the agricultural context. The detections are aligned using 2D occupancy grid mapping. Suvei et al. (2018) proposes a vision-based method to detect protruding objects in front of the agricultural robot. The method fuses data from LiDAR and stereo cameras to generate a dense and accurate point cloud representation of the environment. The point cloud is then used to detect and label the obstacles by applying PointNet (Qi et al., 2017). The work presented in (Skoczeń et al., 2021) proposes an obstacle detection and mapping system for a lawn mower robot based on RGB-D cameras. The semantic mask of the environment obtained on the RGB image is combined with the depth image to project obstacles on 2D occupancy grid. The determined grid is then utilized by the navigation algorithm for obstacle avoidance planning.

Vision-based obstacle detection has been researched for rice farming in paddy fields. The work presented by Qiu et al. (2020) combined YOLOv3 (Redmon and Farhadi, 2018) and deep Simple Online and Realtime Tracking (Deep SORT) (Wojke et al., 2017) to detect and track obstacles in paddy fields using RGB images. The algorithm is applied to RGB images to track moving obstacles in the paddy fields. An obstacle detection algorithm for rice combine harvesters is proposed by Li et al. (2020). The obstacles are detected by a semantic segmentation algorithm that is obtained by applying Network Slimming method (Liu et al., 2017) to ICNet (Zhao et al., 2018).

The lack of labelled datasets for obstacle detection in agriculture poses a major challenge to applying deep learning architectures in the agricultural scene understanding. The dataset presented in (Kragh and Underwood, 2020) contains annotated images, point clouds and navigation data intended for multi-modal object detection. The dataset was collected in various orchard environments and dairy farms in Australia. A large-scale dataset for human detection in an apple orchard and orange grove is introduced in (Pezzementi et al., 2018) and addresses the challenges of occlusion and non-standard poses. A multi-modal dataset for obstacle detection in agriculture is presented in (Kragh et al., 2017). The dataset is collected for the grass-mowing scenario and contains 2 h of raw sensor data, including data from the multiple cameras (stereo camera, thermal camera, web camera, 360 °camera), LiDAR, radar, IMU and GNNS. The dataset is annotated for GPS position and object labels.

This paper focuses on vision-based scene understanding and application in the agricultural environment. The paper presents a framework for combining the detection of multiple scene understanding tasks. The proposed ensemble method is an extension of the author’s previous work on semantic segmentation (Mujkic et al., 2020), anomaly detection and object detection (Mujkic et al., 2022). Deep-learning based-algorithms for semantic segmentation, object detection and anomaly detection are trained individually. The model for semantic segmentation is based on SegNet (Badrinarayanan et al., 2017) architecture and trained to detect the field area in an image. In the case of agricultural vehicles driving in the field, the detected field area is considered a broad region of interest for detecting potential collision risks. The YOLOv5 (Jocher, 2020) object detector is applied to detect and classify objects belonging to seven agriculture-specific classes: ‘tractor’, ‘combine’, ‘trailer’, ‘combine header’, ‘baler’, ‘square bale’ and ‘round bale’. The anomaly detector based on the semi-supervised convolutional autoencoder is used to identify other objects that do not belong to previously mentioned classes and assign them with the class ‘unknown’. The detection results from different algorithms are combined at inference time, and the proposed ensemble method is independent of underlying algorithms for each task. In the proposed ensemble method, detections from the object detector and anomaly detector are combined, and a segmentation map of the field is used to identify if objects are inside the field area or not.

The main contributions of this paper are the following:

• An ensemble method for combining object detection and anomaly detection with a semantic segmentation map of the agricultural field.
• Evaluation of the algorithms and ensemble method on agricultural dataset

The paper is structured as follows. The individual models used in the paper are introduced in Section 2 and the proposed ensemble method is presented. In Section 3, the performance of the proposed ensemble method is evaluated. This is followed by the conclusion in Section 4.

## 2 Materials and methods

This section briefly describes datasets and models used for semantic segmentation, object detection and anomaly detection. This is followed by the description of the proposed ensemble method.

### 2.1 Datasets

The lack of labelled datasets poses a major challenge to applying deep learning architectures in the agricultural scene understanding. In order to address this challenge, the models were trained on several datasets collected specifically for the operation of agricultural vehicles in multiple agricultural fields. The overview of the datasets is provided in Table 1.

**TABLE 1:** Overview of datasets.

Task		Annotation	Classes	# Images	Resolution
Semantic segmentation		Pixel level	field, other	818	2048 × 770
Object detection		Bounding box	tractor, combine, trailer, combine header, baler,	14.3k	Varying
			square bale, round bale		
Anomaly detection	Normal data	None	None	1408	3206 × 1898
	Abnormal data	Pixel level	anomaly	300	3206 × 1898
Evaluation		Bounding box	tractor, combine, trailer, combine header, baler,	7.9k	3206 × 1898
			square bale, round bale, human, truck, car		

The dataset for semantic segmentation was collected during the harvester’s operation in the fields. The dataset consists of 818 RGB images with the corresponding pixel-wise labelled ground truth images annotated for classes ‘field’ and ‘other’.

The dataset for object detection consists of 14.3k RGB images annotated with 2D bounding boxes. The annotated classes are: ‘tractor’, ‘combine’, ‘trailer’, ‘combine header’, ‘baler’, ‘square bale’, and ‘round bale’.

For the training of a semi-supervised autoencoder, a dataset consisting of 1408 normal images and 300 images with anomalies annotated at the pixel level is used. The images were collected for the harvesting scenario over 9 days.

The dataset for evaluation of the ensemble method consists of 7.9 k images collected by two agricultural vehicles over 13 days. The annotated classes include agricultural vehicles and implements, road vehicles, static objects like bales, and humans.

### 2.2 Semantic segmentation model

**FIGURE 1:**
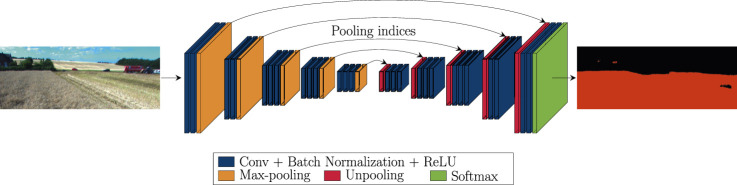
Illustration of SegNet architecture applied to a field area segmentation in the agricultural scene. The input of the network is an RGB image of a field scene. The output is the corresponding segmented image.

This paper applies a deep architecture SegNet to solve semantic segmentation tasks for the agricultural environment. The architecture of the network is illustrated in Figure 1. The SegNet architecture was selected because it allows for the efficient storage of encoder feature maps. In contrast to architecture presented in (Ronneberger et al., 2015) that stores the full encoder network feature maps, SegNet stores the max-pooling indices of the feature maps and uses them to upsample feature maps in the decoder network. The work presented in (Noh et al., 2015) uses a similar technique for upsampling in the decoder network. However, the proposed architecture has a significantly larger number of parameters and longer training and inference time. Furthermore, SegNet is a fully convolutional network that can take images of any size as input. SegNet architecture consists of symmetrical encoder and decoder networks. The topology of the encoder network corresponds to the first 13 convolutional layers of the VGG16 (Simonyan and Zisserman, 2015) network. Each encoder layer in the network is composed of convolutions, batch normalizations, and Rectified Linear Unit (ReLU) nonlinearity, followed by a non-overlapping max-pooling layer. The max-polling indices of the feature map in the encoder are stored and used to upsample the corresponding feature map in the decoder network. The layers in the decoder are composed of unpooling layer, convolutions, batch normalization and ReLU nonlinearity. Finally, the upsampled feature maps are convolved to produce dense feature maps. The feature map produced by the decoder network is fed to a soft-max classifier that generates class probabilities for each pixel. For N classes, the output of the softmax classifier is an N-channel image of pixel-wise probabilities. For each pixel, the predicted class is the one with the highest probability.

### 2.3 Object detection model

The YOLOv5m was chosen for the object detection task. YOLOv5 is a real-time object detector composed of a backbone network, neck, and detection head. The backbone network extracts the input image features. In Yolov5 the Cross-Stage Partial Connections (CSP) network (Wang et al., 2020) is used as the backbone. Path Aggregation Network (PANet) (Liu et al., 2018) is applied to extract feature pyramids. The detection head generates the final output vector of class probabilities, objectness score and bounding boxes. YOLOv5 uses the same detection head as proposed in YOLOv3. The activation function in hidden layers is Sigmoid Linear Units (SiLU), while the final detection layer uses the sigmoid activation function.

### 2.4 Anomaly detection

**FIGURE 2:**
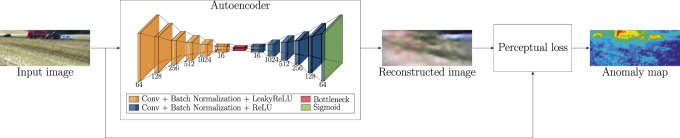
Illustration of the anomaly detection method. The convolutional autoencoder is applied for the image reconstruction task. The input of the network is an RGB image of a field scene. The output is the corresponding reconstructed image. The anomaly map is generated by applying relative-perceptual- L1 loss to the input image and the corresponding reconstructed image.

In this paper, a semi-supervised convolutional autoencoder is used to detect anomalies in field scene images. The anomaly detection concept and the architecture of the network are illustrated in Figure 2. The network consists of encoder and decoder parts with six convolutional layers. The encoder network has an increasing number of filters (64, 128, 256, 512 and 1024), while the decoder has a decreasing number of filters (1024, 512, 256, 128 and 64). The encoder network and decoder network share a bottleneck with 16 channels. Each convolutional layer, except the final layer, is followed by batch normalization and LeakyReLU as an activation function. The final convolutional layer is followed by sigmoid activation.

Autoencoders for anomaly detection are often trained in an unsupervised manner. Using normal data without anomalies to train the autoencoder enables the model to learn to reconstruct the normal data instances from low-dimensional feature space. However, the anomalies are much harder to reconstruct from the same low-dimensional feature space. Therefore, they result in significantly larger reconstruction error than normal data. This difference in the reconstruction error can then be used to identify anomalies.

The loss function of semi-supervised autoencoder consists of two terms that handle normal and abnormal data. The loss function is given by:

$$L\left(x,y\right)=\frac{1}{N}\overset{N}{\underset{i}{\sum }}\|x_{i}-y_{i}{\|}_{2}+\text{max}\left(0,a_{0}-\frac{1}{M}\overset{M}{\underset{i}{\sum }}\|x_{i}-y_{i}{\|}_{2}\right),$$ (1)

where *N* is the total number of normal pixels in the image, *M* is the total number of abnormal pixels, *x*_*i*_ is *i*th pixel value in the input image, and *y*_*i*_ is the value of the corresponding pixel in the reconstructed image. Threshold *a*_0_ is the margin that separates normal and abnormal pixels. The optimal value is determined experimentally as *a*_0_ = 0.2.

Relative-perceptual-L1 loss (Tuluptceva et al., 2019) between the input and reconstructed images is used to generate an anomaly map.

### 2.5 Ensemble method

**FIGURE 3:**
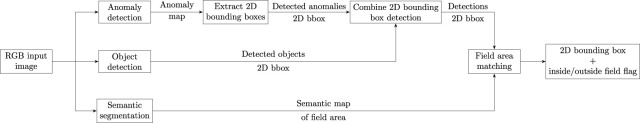
Diagram of proposed ensemble method.

Figure 3 illustrates the proposed ensemble method. The method consists of several steps for combining the detections of individual models. First, anomaly maps are processed to extract the bounding boxes for detected anomalies. Next, the objects detected as anomalies that have also been detected and classified by the object detector are removed. The remaining anomalies are assigned a class ‘unknown’ and added to the list of detected objects. For each detected object, the bounding boxes are compared with a segmentation map of the field to determine if the object is inside the field or not. The following sections describe each step in more detail.

#### 2.5.1 Extraction of bounding boxes for detected anomalies

The first step in converting anomaly detections to bounding box representation is thresholding the anomaly map to obtain a binary anomaly map. The optimal threshold is a constant value of 1.4. The binary anomaly map is processed by connected components labelling algorithm to extract components that exceed a certain area. A contour extraction algorithm is applied to extract the boundaries of extracted components and subsequently use them to compute the bounding rectangles for each.

**FIGURE 4:**
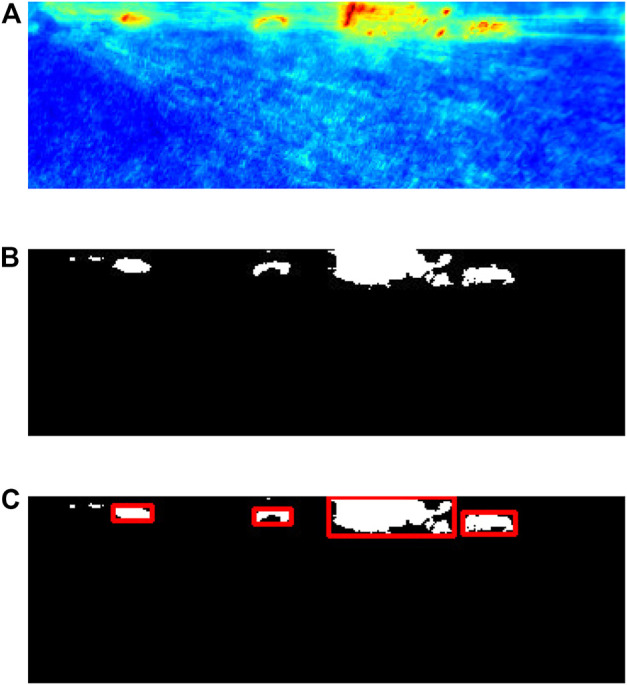
Anomaly detection processing steps **(A)** Anomaly map obtained from autoencoder. **(B)** Binary anomaly map. **(C)** Extracted bounding boxes for detected anomlies.

An example of the procedure is shown in Figure 4. Figure 4A shows an example of an anomaly map generated by the autoencoder. The binary anomaly map obtained after thresholding is shown in Figure 4B. Figure 4C shows the results of applying the connected components algorithm and computed bounding boxes.

#### 2.5.2 Combining of 2D bounding box detections

After the conversion of anomaly detections to bounding box representation, they are combined with detections from the object detector. Some objects in the images are detected by both anomaly detectors and object detectors. In those cases, object detections with class labels are prioritized. Therefore, the bounding boxes of detected anomalies are compared with detected objects, and the anomalies that intersect with bounding boxes of detected objects with more than 30% of their area are ignored. The anomaly detections that remain are assigned class ‘unknown’ for consistency with detections from object detectors.

**FIGURE 5:**
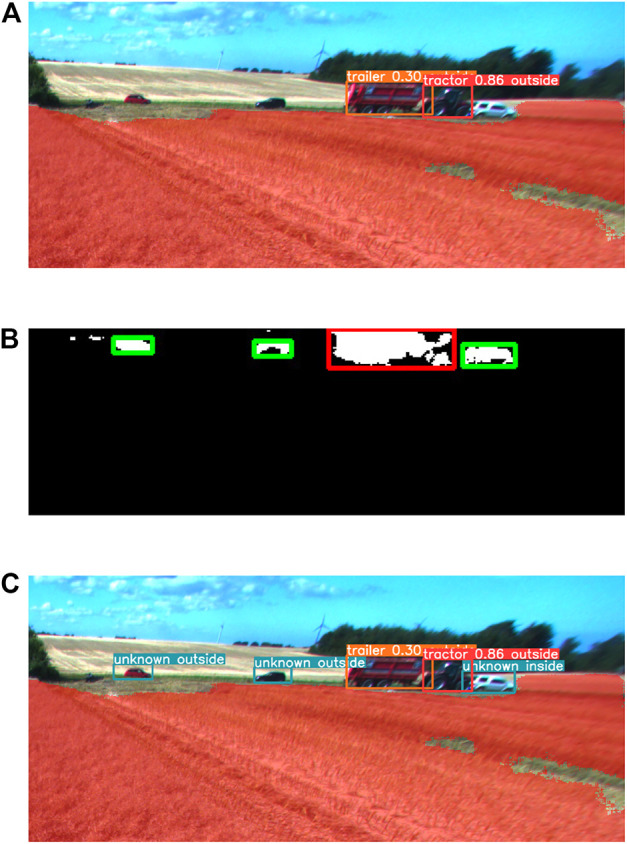
Combining detections from object detector and anomaly detector **(A)** Object detection results. **(B)** Anomaly detection results in bounding box representation. Green bounding box indicates object that has not been detected by object detector. **(C)** Combined detection results from object detector and anomaly detector.

Figure 5 illustrates combining the detections from the object detector and anomaly detector. The detections from the object detector are shown in Figure 5A. The algorithm detected two objects successfully and failed to detect three objects. Figure 5B shows detected anomalies for the same image. The green boxes indicate objects that have not been detected by the object detector and need to be included in detections as anomalies. Figure 5C shows combined detection results from the object detector and anomaly detector. Two objects are detected and classified by the object detector, while three objects are detected by the anomaly detector and assigned class ‘unknown’.

#### 2.5.3 Field area matching

The combined detections from the anomaly and object detector are assigned an additional label indicating whether they are inside or outside the field. The semantic map of the field provided by the semantic segmentation module labels the pixels corresponding to the surrounding field of the object. This information is combined with the location of individual bounding boxes in the images to determine whether the object is inside the field. The procedure involves a few steps. First, the detected object’s bounding box is dilated by 20% in the *x* and *y*-direction. Then, the bottom half of the region between the original bounding box and dilated bounding box is selected. If more than 10% of this region’s area is segmented as a field, the object is considered to be within the field. Otherwise, the object is considered to be outside the field.

**FIGURE 6:**
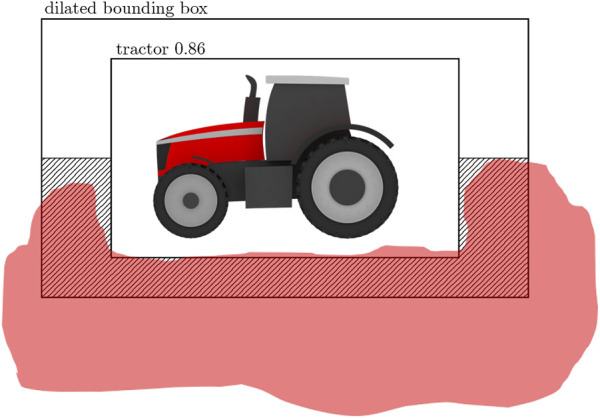
Illustration of method for inside/outside of field classification for detected objects. The hashed region indicates region for evaluating pixels segmented as field area. The red region is an example of segmented field area.

An example illustrating the method is shown in Figure 6. In this example, a tractor is detected by the object detector, and its bounding box is dilated. The hashed region indicates the bottom half of the region between the original box and dilated box. The red area illustrates an example of an area segmented as a field by the semantic segmentation module. In this example, more than 10% of the hashed region area is segmented as a field; therefore, the object will be classified as inside the field.

## 3 Results

The training hyperparameters for individual models are listed in Table 2. The performance of the method is evaluated on a dataset of 7.9k images of agricultural scenes. The object and anomaly detector results are reported using a confusion matrix. In addition, the classification of objects as being inside or outside of the field is evaluated qualitatively.

**TABLE 2:** Training parameters.

	**Semantic segmentation**	**Object detection**	**Anomaly detection**
Epochs	623	300	500
Learning rate	(0.01, 0.001)	(0.01, 0.1)	1e − 5
Optimizer	SGD	SGD	Adam
Momentum	0.9	0.937	(0.9, 0.999)
Weight decay	0.0001	0.0005	0
Batch size	8	64	32
Image size	1024 × 385	640 × 640	800 × 160

For the purpose of evaluating the performance of object detector and anomaly detector, objects in the testing dataset are considered to belong to a single general class ‘object’. For the object detector, only detections with a confidence score above 0.25 are considered. The IoU threshold for ground truth and bounding box detections is selected as 0.45. The results shown in Table 3 indicate that combining object detector and anomaly detector increases the number of detected objects from 12759 to 13547. However, anomaly detector also introduces a substantial number of false-positive detections.

**TABLE 3:** Perfromance evaluation of object detector and anomaly detector. TP is the number of true positives, FN is the number of false negatives and FP is the number of false positives.

	**Object detector**	**Object detector and anomaly detector**
TP	12759	13547
FN	6894	6106
FP	7204	13299

**FIGURE 7:**
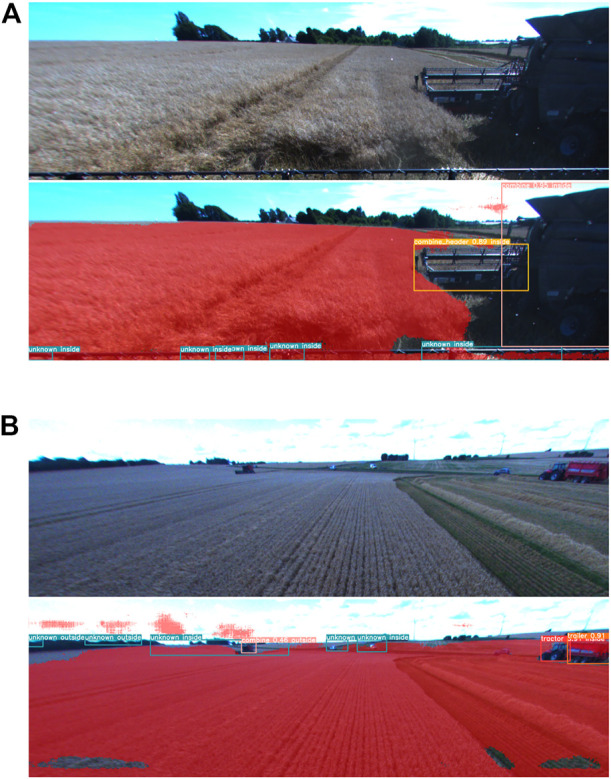
Detection examples for ensemble method **(A)** Parts of combine header being detected as anomalies. **(B)** Shelterbelt in distance being detected as multiple anomalies.

Examples in Figure 7 provide some insights into the source of these false-positive anomalies. Figure 7A shows an example image where parts of the vehicle’s combine header are present at the bottom of the image. Since the training images of normal operating conditions did not have this, the parts of combine header are detected as multiple anomalies. Another example is given in Figure 7B. Here distant objects are correctly detected as anomalies. However, on the left-hand side of the image, the shelterbelt is detected as multiple anomalies. In this case, the training dataset for the anomaly detector had images depicting mostly crop field areas; therefore, parts of the shelterbelt are reconstructed poorly and falsely detected as anomalies. It is worth mentioning that having false-positive anomaly detections is acceptable from a safety perspective.

**FIGURE 8:**
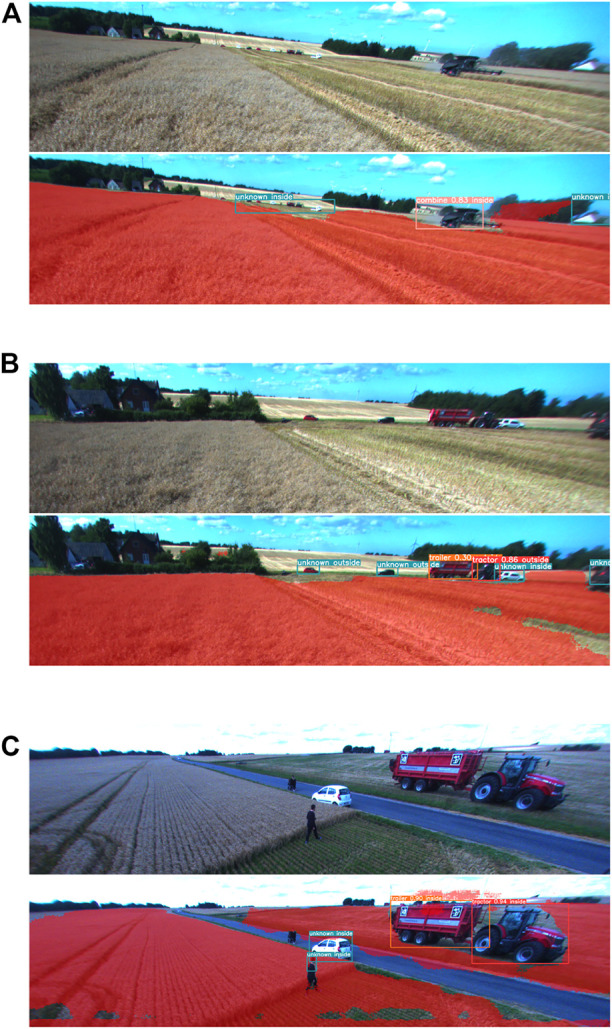
Detection examples for ensemble module **(A)** Group of distant vehicles detected as an anomaly. **(B)** Object not detected by object detector is detected as an anomaly instead. **(C)** Detected objects correctly classified as being inside or outside of the field.

Figure 8 provides further examples of the ensemble method’s performance. Figure 8A shows an example where a group of distant vehicles in the background is detected as a single anomaly. A farmhouse is also detected as an anomaly on the right-hand side of the image. An example in Figure 8B shows cars not being detected by an object detector. However, the anomaly detector was able to detect them. Figure 8C shows an example of vehicles and persons being detected by the object detector and anomaly detector.

The classification of detected objects as inside/outside of the field by the proposed method was evaluated in the example images. In Figure 7A all detected objects are correctly classified as being inside of the field. Figure 7B shows that false-positive anomaly detections of shelterbelt are classified as being outside of the field, while anomaly detected in the same area as combine harvester is classified as being inside of the field. These results are in agreement with the segmented field area. The ‘combine’ detection is misclassified as being outside of the field due to the poor segmentation of the field around it. In Figure 8A detections are classified correctly as being inside of the field despite the large areas around them not being classified as field areas. This is due to the large size of the bounding boxes, which resulted in a greater area considered in the calculation of field segmentation overlap. Figure 8B shows a car on the right-hand side of the image correctly classified as being inside the field. The two cars to the right are classified as being outside the field. The detected tractor is falsely classified as being outside of the field.

Both Figures 8A, B depict a similar scene from different distances. The group of cars detected as an anomaly in the first figure and two cars in the second figure are parked at the edge of the field. However, this is not clearly visible in these images, and therefore the correct classification might depend on the distance and the camera’s angle. This illustrates one of the shortcomings of using a single camera sensor. The example in Figure 8C shows three vehicles correctly classified as being inside of the field. Moreover, there is one pedestrian inside the field and one on the road. The pedestrian on the road was not detected and the one in the field is correctly classified as being inside the field.

## 4 Conclusion

The proposed ensemble method combines the three scene understanding approaches to provide contextual information about the detected object and anomalies. The performance of the ensemble method is evaluated for detecting objects in agricultural scene images. The results showed that combining object detection with anomaly detection increased the number of detected objects in the test dataset from 12759 to 13547. It was observed that the anomaly detection introduced false-positive detections, and a short discussion was provided. The semantic field map has been combined with the detections to provide additional information regarding the detected object’s location. However, the proposed approach is sensitive to segmentation accuracy and the camera angle.

Future work will investigate other approaches for combining semantic segmentation with object detection.

## Data Availability

The raw data supporting the conclusions of this article will be made available by the authors, without undue reservation.
